# Advances in ADHD, An Opportunity for Learning and Further Research

**DOI:** 10.3390/brainsci15111156

**Published:** 2025-10-28

**Authors:** Joseph Sadek

**Affiliations:** Department of Psychiatry, Faculty of Medicine, Dalhousie University, Halifax, NS B3H 4R2, Canada; joseph.sadek@nshealth.ca

MDPI’s open-access journal *Brain Sciences* has published a new Special Issue focused on advances in ADHD. This Special Issue captured important areas in the epidemiology, risk factors, diagnosis, and management of ADHD. It also highlighted practical considerations in the functional impairments and comorbidities of ADHD.

It was interesting to learn about the results of the prevalence and incidents of ADHD in published Canadian data. The overall estimate in children and adolescents was 8.6% and in adults was 2.9%. Estimate varied by age, gender, province, and time, but overall, the Canadian ADHD prevalence estimate was comparable with the worldwide estimates, particularly in adults (Contribution 1).

One landmark study in this Special Issue was a systematic review and meta-analysis of psychosocial interventions for ADHD. This evidence supports a recommendation for cognitive behavioral therapy for adults with ADHD. The evidence also supports caregiver intervention for children, but not for preschoolers. There were not enough data to provide recommendations for other types of psychosocial interventions (Contribution 2).

This Special Issue also provided guidance on the definition of functional impairment in ADHD and tools for the measurement of functional impairment, with a conclusion of the proposal of a paradigm shift in looking at the relationship between symptom response and function. The assessment of functional impairment is an important task in the diagnosis and management of ADHD (Contribution 3).

Risk factors and etiology were captured by more than one study in this Special Issue.

One study on developmental risk and adversity experience in relation to the clinical profile of ADHD showed significant findings: the average number of comorbidities increased with the severity of ADHD; the average number of risk and adversity factors increased with the severity of ADHD; the number of risk and adversity factors was positively associated with the number of comorbidities; and, finally, the level of education was negatively associated with the number of risk factors and number of comorbidities (Contribution 4).

An additional study focused on risk and adversity factors, such as birth complications, delayed milestones, bullying, family issues, sexual abuse, physical abuse, poverty, trouble with the law, and completing grade 12 education, in adult patients with comorbid ADHD, with binge eating disorder (BED), and with borderline personality disorder (BPD). The results suggested that there was a significant association between a principal diagnosis of ADHD with comorbid BPD and BED and having four or more risk factors. This study suggested that specific risk factors such as family issues, bullying, poverty, trouble with the law, and physical abuse are associated with these specific comorbidities. ADHD patients without comorbid, borderline personality disorder and binge eating disorder have less risk factors compared with those patients with comorbidities (Contribution 5).

A study from the Netherlands suggested that social media use in adolescence with ADHD is quite problematic. The researchers proposed a social mechanism and a biological mechanism for the link between ADHD and social media use. The debate about the hours spent watching television as a child and the attention problems in adolescents continues, but several studies support this hypothesis (Contribution 6).

Clinicians who treat ADHD may benefit from further learning regarding malingering and stimulant medication abuse, misuse, and diversion. Clearly, the problem of malingering and stimulant use disorder requires further research; this issue constitutes a public health concern since stimulant medications have major adverse effects and addictive potential.

This article encourages further research into the potential use of objective measures of ADHD testing based on neuroimaging methods or neuropsychological testing to avoid the feigning of ADHD symptoms (Contribution 7).

A Canadian study by Seal and colleagues on the impact of COVID-19 on physical activities in families managing ADHD identified a vital need to support physical activity opportunities during high-stress situations in families, managing ADHD to buffer against diminishing mental well-being (Contribution 8).

Attention was drawn to working memory, particularly in the relationship with vocabulary in normal development versus in children and adolescence with ADHD in a study by Rodriguez-Martínez et al. (Contribution 9).

Working memory deficits in children and adolescents with ADHD were also correlated with the visuospatial sketchpad (VSS) during testing when a specific neuropsychological test (K-BIT) was used (Contribution 9).

Cai and colleagues compared children with ADHD with typically developed children to examine the cognitive control over working memory capture of attention in both groups. The results suggested that a significant difference was found between the two groups and that attentional capture may be improved by multisensory working memory encoding (Contribution 10).

Handelman and Sumiya suggested that further research is required to define clinical tolerance for stimulants in ADHD and to provide guidance on identifying and managing tolerance in clinical practice (Contribution 11).

Despite the advances in ADHD research, there are several areas that require further research in this field. The diagnostic accuracy of ADHD, rating scales for the follow-up of patients with ADHD, and long-term studies on the safety of ADHD medications are examples of future research directions. 

I conclude by stating that this Special Issue provides a unique learning opportunity for clinicians and researchers who are interested in the field of ADHD.
